# A novel, nature-based alternative for photobioreactor decontaminations

**DOI:** 10.1038/s41598-019-39673-6

**Published:** 2019-02-27

**Authors:** Lisa Krug, Armin Erlacher, Gabriele Berg, Tomislav Cernava

**Affiliations:** 1 ACIB GmbH, Petersgasse 14, 8010 Graz, Austria; 2 Institute of Environmental Biotechnology, Graz University of Technology, Petersgasse 12, 8010 Graz, Austria

## Abstract

Large-scale microalgae cultivations are increasingly used for the production of animal feed, nutritional supplements and various high-value bioproducts. Due to the process size and other limitations, contaminations of microalgae fermentations with other photoautotrophic microorganism are frequently observed. In the present study, we explored the applicability of 5-isobutyl-2,3-dimethylpyrazine for the removal of contaminating microalgae from industrial photobioreactors. In order to select a representative microbial population for susceptibility experiments, reactor samples were obtained from a multi-stage cultivation process. Assignments of 18S rRNA gene fragment amplicons indicated that *Haematococcus*, *Chlorella*, and *Scenedesmus* were the three most frequently occurring microalgae genera in the selected reactors. Following the isolation of representative algae cultures, susceptibility tests were conducted with the antimicrobial pyrazine. It was demonstrated that all isolated contaminants are highly susceptible to the bioactive compound. The highest tolerance towards the alkylpyrazine was observed with *Scenedesmus vacuolatus*; solutions with 1.66% (v/v) of the active compound were required for its deactivation. Further tests with the vaporized pyrazine showed consistent reductions in the viability of treated microalgae. This pilot study provides evidence for the applicability of a novel, nature-based alternative for bioreactor decontaminations.

## Introduction

The industrial relevance of microalgae as production systems for valuable bioproducts and as promising feedstocks for biofuel production is constantly increasing^1–3^. Modern cultivation processes are optimized for high-yield production and utilize various eukaryotic whole-cell systems for a broad spectrum of bioproducts. *Chlorella*, *Dunaliella*, and *Scenedesmus* are the most commonly employed genera in production-scale photobioreactors with production capacities of up to 3,000 tons per year; various other photoautotrophs are used for more specific fermentations^4^. Microalgae cultivation is considered as sustainable, because it only requires solar energy under photoautotrophic conditions to produce a range of highly valuable products, including pharmaceuticals, fertilizers and food supplements^5^. Industrial-scale cultivations under conditions required for bulk material production are mostly based on open pond systems. In contrast, the production of high-valuable compounds from microalgae is mainly done on the basis of closed photobioreactors in order to reproduce production conditions, increase the control of cultivation variables and reduce the risk of contaminations^6,7^. Under both process conditions, one of the main constrains for an efficient cultivation of microalgae is the potential contamination with biological pollutants, such as bacteria, fungi, zooplankton or other undesirable microalgae^5,8–10^. Some contaminants like *Poterioochromonas* spp. (*Chrysophyta*) not only compete for nutrients with the cultivated algae, but also impair their growth by toxin production^11^. This can even result in a collapses of the cultivation batch as shown by Ma and colleagues^12^. In order to reduce or prevent the negative impact of contaminations, a range of viable strategies have been implemented so far. Common strategies include early harvesting of the product to avoid serious biomass losses, or the use of a number of chemical, biological and physical treatments^13^. As a safety precaution in-between cultivation processes, photobioreactors are often emptied and decontaminated with different treatments^14–16^. Common decontamination procedures include rinsing of the reactors with sodium hypochlorite or the application of hydrogen peroxide^17,18^. Here, the low stability and the high reactivity of the disinfectant are often disadvantageous for the process environment. Due to various safety reasons and the instability and reactivity of the currently employed decontaminants, efficient alternatives could improve industrial-scale microalgae cultivations.

One so far untapped environmentally friendly alternative for bioreactor decontamination could be the application of naturally occurring antimicrobials that are emitted by highly competitive microorganisms. Studies with *Paenibacillus polymyxa* isolates from plant roots and endophytes from inner plant tissues showed that they produce various highly antimicrobial volatile organic compounds (VOCs)^19^. Among other bioactive compounds in their volatilomes, alkylpyrazines were identified as carriers of antimicrobial effects of the beneficial bacteria. These isolates not only showed high inhibition efficiency against plant pathogenic fungi, but also the potential to inhibit potential human pathogens^20^. These results imply that mimicking the bioactive volatilome of *P. polymyxa* is a promising strategy to control diverse microbial contaminations. This strategy was already applied for the decontamination of biological surfaces and to reduce contamination in processed meat products^21,22^ and is patented for specific applications^23^. In the present study, 5-isobutyl-2,3-dimethylpyrazine was employed, because of its similar effect to the pyrazine mixture emitted by *P. polymyxa* GnDWu39, which is a highly competitive biocontrol agent^24^. We wanted to find out if this alkylpyrazine derivative is effective against representative microalgae contaminations at concentrations that were previously shown to be sufficient to treat bacterial contaminants^22^. In order to evaluate its applicability, the model pyrazine was evaluated by implementing two different application strategies that could also find application in full-size bioreactors.

## Results

### Diversity of the eukaryotic community in photobioreactors

**Figure 1:**
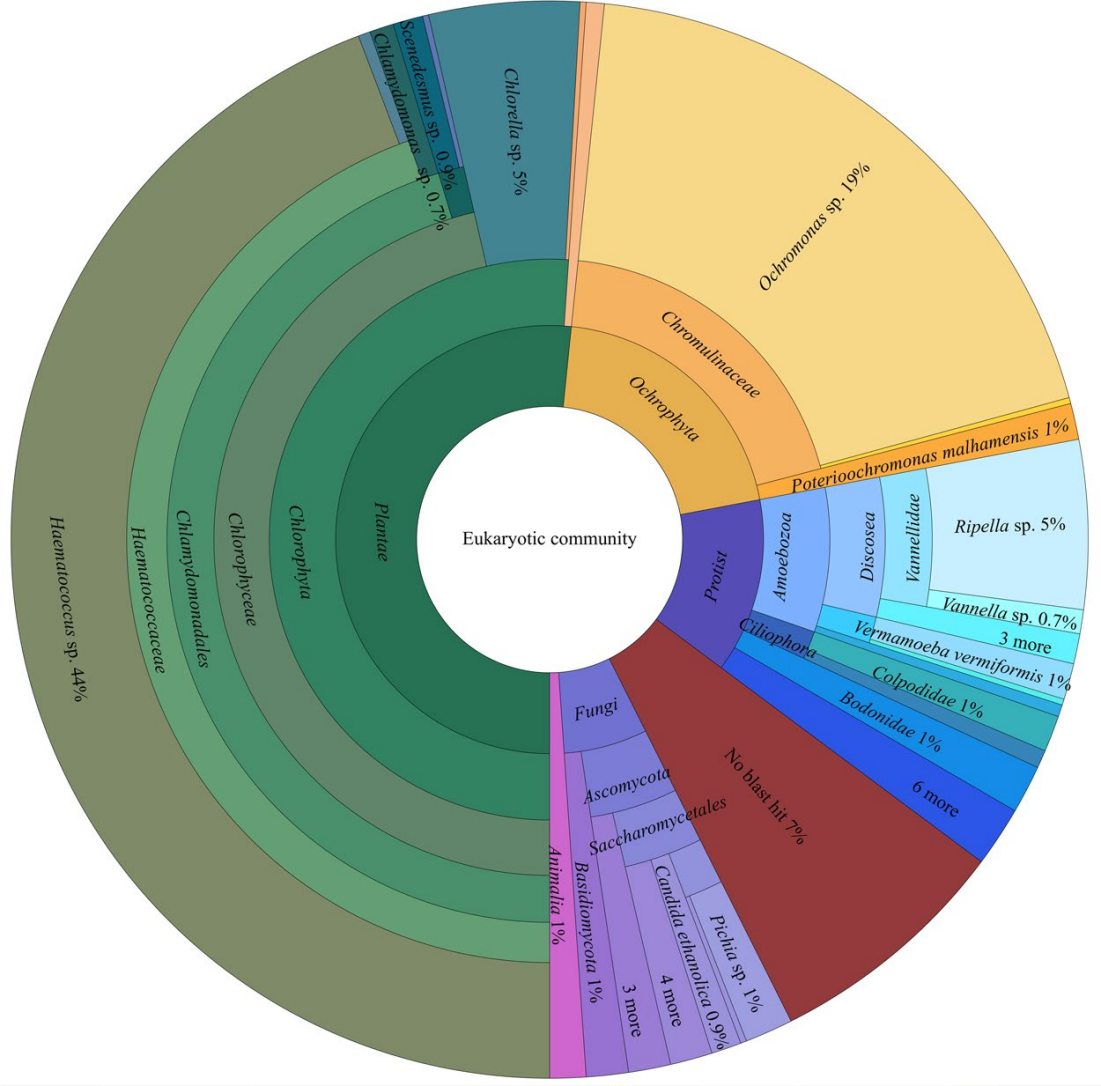
Composition of the eukaryotic reactor community in an industrial *Haematococcus* sp. cultivation process. Taxonomic information is based on 18S rRNA gene sequence analyses and assignments within the NCBInt database. Each circle represents a different taxonomic rank (innermost circle: kingdom; outermost circle: genus). The percentage represents the relative number of OTUs for each taxonomic group over the whole cultivation process.

The overall dataset that was obtained by sequencing of 18S rRNA gene fragments in eight reactor samples contained a total of 4,992,008 reads. After removal of bacterial and archaeal sequences 4,886,808 reads remained, and were clustered in 554 operational taxonomic units (OTUs). The overall structure of the eukaryotic community is shown in Table 1. In the filtered dataset, a major proportion consisting of 4,512,85 reads (92.3%) was clustered in 245 OTUs (44.3%) and assigned to *Haematococcus* (genus level); 1,871 reads (0.04%) were clustered in five OTUs (0.9%) and assigned to *Scenedesmus* and 37,986 reads (0.78%) in 25 OTUs (4.5%) to *Chlorella* (Fig. 1). In total 286 OTUs (51.6%) were assigned to *Plantae* and included seven genera in addition to the mentioned microalgae. 113 OTUs (20.4%) were assigned to *Chromista* sharing 140,545 reads and further classified as three different genera including *Ochromonas* (106 OTUs, 140,519 reads), *Poterioochromonas* (6 OTUs, 18 reads) and *Spumella* (1 OTU, 8 reads). Moreover, 21 fungal species were identified (35 OTUs, 6.3%; 531 reads). *Protista* were represented by 73 OTUs (13.2%) and further identified as 23 different genera, whereby the most abundant genus was *Ripella* with a total amount of 28 OTUs and 160,259 reads; a total of 190,531 reads was assigned to *Protista*. Six OTUs (1.1%) were assigned to *Animalia* (1,289 reads, assigned to four different species). In addition, 41 OTUs (7.4%) remained unassigned (579 reads). Overall, the unambiguously identified fraction of contaminating microorganisms in the photobioreactors was 7.7% based on read numbers.

**Table 1:** Biodiversity in photobioreactors assessed with 18S rRNA gene fragment amplicon sequencing.

		OTU count		Read count	
		absolute	rel. [%]	absolute	rel. [%]
***Animalia*** 6 OTUs (1.08%) 1,284 reads (0.03%)	*Rotifera*	4	0.72	1,282	0.03
	*Anthropoda*	1	0.18	1	<0.01
	*Chordata*	1	0.18	1	<0.01
***Chromista***	*Ochrophyta*	113	20.40	140,545	2.88
***Fungi*** 148 OTUs (26.71%) 141,076 reads (2.89%)	*Ascomycota*	28	5.05	461	0.01
	*Basidiomycota*	7	1.26	70	<0.01
***Plantae*** 268 OTUs (51.62%) 4,553,333 reads (93.18%)	*Chlorophyta*	283	51.08	4,553,107	93.17
	*Angiosperm*	2	0.36	216	<0.01
	*Streptophyta*	1	0.18	10	<0.01
***Protista*** 73 OTUs (7.40%) 190,531 reads (3.90%)	*Amoebozoa*	46	8.30	178,377	3.65
	*Apicomplexa*	1	0.18	27	<0.01
	*Ciliophora*	9	1.62	696	0.01
	*Euglenophyta*	4	0.72	1167	0.02
	*Euglenozoa*	8	1.44	9,867	0.20
	*Excavata*	1	0.18	39	<0.01
	*Heterokontophyta*	1	0.18	270	0.01
	*Perclozoa*	1	0.18	8	<0.01
	*Rhizaria*	2	0.36	8	<0.01
No blast hit		41	7.40	579	0.01

The community structure of eukaryotic taxa within an industrial scale photobioreactor was assessed with the QIIME 1.9.0 pipeline and BLAST searches within the NCBI nucleotide database.

### Selection of representative microalgae isolates for decontamination experiments

**Figure 2:**
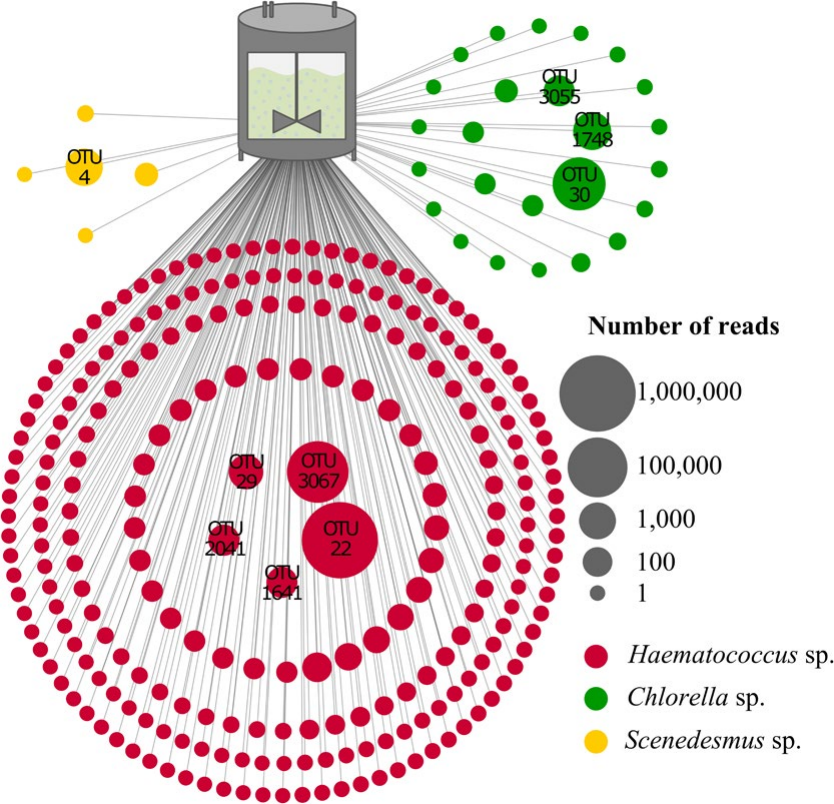
Schematic visualization of the predominant microalgae subpopulation in an industrial cultivation process. The pictured OTUs were assigned to *Chlorella* (green), *Haematococcus* (red) and *Scenedesmus* (yellow). Node sizes correspond to the number of reads that was assigned to each OTU. All OTUs with more than 100 reads were labeled and subjected to manual BLAST searches against NCBInt. Taxonomic assignments at species level are included in Table S1.

Based on the microbiome analysis of the multi-stage cultivation process, three microalgae isolates were selected that represent the reactor’s algae population. The genera *Haematococcus, Scenedesmus*, and *Chlorella* together accounted for 93.1% of the reads and 49.7% of the OTUs in the analyzed reactors (Fig. 2). Manual BLAST searches assigned the representative OTU sequences of abundant (>100 reads) *Haematococcus* hits to two *H. lacustris* entries in the NCBInt database (Supplementary Table S1). Representative *Chlorella* sequences for OTUs with the same read threshold were assigned to *C. vulgaris* and two additional *Chlorella* sp. entries. Only one *Scenedesmus* OTU met the read threshold and the representative sequence was assigned to *S*. *vacuolatus*. Accordingly, reactor isolates assigned to *H. lacustris*, *C. vulgaris*, and *S. vacuolatus* were selected for the decontamination efficiency experiments. The identity of respective isolates was confirmed by Sanger sequencing of the 18S rRNA gene fragment.

### Microalgae treatments with liquid alkylpyrazines

**Figure 3:**
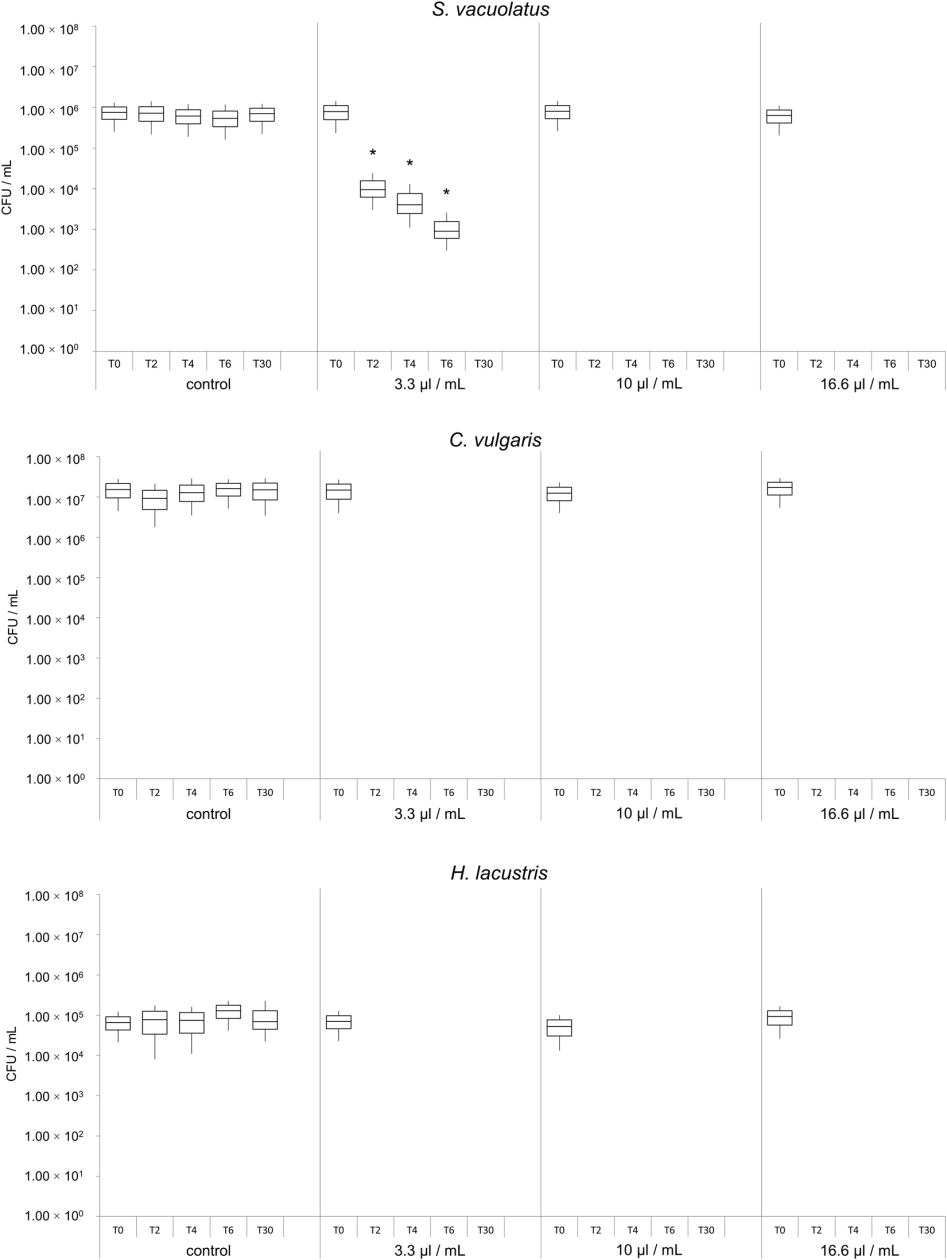
Cultivation-based quantification of viable *S. vacuolatus* (**a**), *C. vulgaris* (**b**) and *H. lacustris* (**c**) cells after treatment with liquid pyrazine. Bars represent the number of viable microalgae cells. Samples were treated with different pyrazine concentrations as indicated in the graph and analyzed at different time points; T0: before treatment; T2: after two hours of incubation; T4: after four hours of incubation; T6: after six hours of incubation; T30: after 30 hours of incubation. Asterisks represent significant differences in cell viability as evaluated with Student**’**s paired t-test at *p* < 0.01.

The application of liquid 5-isobutyl-2,3-dimethylpyrazine in microalgae cultures was highly efficient and led to significant reductions of cell viability for all three treated algae species. In case of *S. vacuolatus*, treatments showed mean reduction rates of 98.2% after two hours, 99.0% after four hours, 99.8% after six hours, and 100% after 30 hours of incubation (Fig. 3a). This was observed with the lowest tested alkylpyrazine concentration (v/v) of 3.3 µL/mL. Higher pyrazine concentrations of 10.0 µL/mL and 16.6 µL/mL led to a reduction rate of 100% after two hours of incubation. For *C. vulgaris* and *H. lacustris* a reduction rate of 100% was already observed after two hours of incubation with each of the tested concentration (3.3 µL/mL, 10 µL/mL and 16.6 µL/mL; Fig. 3b,c; Supplementary Table S2).

### Exposure of microalgae to vaporized alkylpyrazines

**Figure 4:**
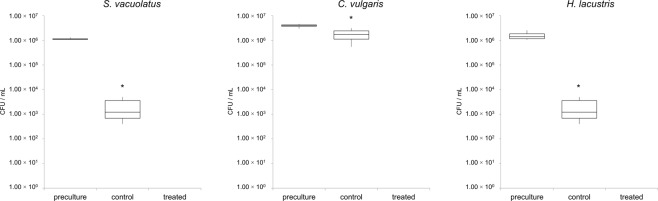
Quantification of viable *S. vacuolatus* (**a**), *C. vulgaris* (**b**) and *H. lacustris* (**c**) cells after treatment with vaporized 5-isobutyl-2,3-dimethylpyrazine. Bars represent CFU counts following 5 h of exposure to the alkylpyrazine in sealed flasks. Asterisks represent significant differences in cell viability as evaluated with Student’s paired t-test (*p* < 0.01).

The number of viable cells was significantly reduced for all tested microalgae after the desiccation procedure. The average reductions for *C. vulgaris* (20.0%), *H. lacustris* (90.3%) and *S. vacuolatus* (99.8%) were highly dissimilar (Supplementary Table S3). Treatment of microalgae with vaporized 5-isobutyl-2,3-dimethylpyrazine resulted in similar reductions as already observed with fluid applications. For *C. vulgaris*, which had the highest recovery rate after the dehydration step, a decrease of 100% in CFU was observed. The same reduction rates were achieved when *S. vacuolatus* and *H. lacustris* were treated; after five hours of incubation a reduction rate of 100% in cell viability was observed for all microalgae isolates (Fig. 4).

### Microscopic visualization of the effects

**Figure 5:**
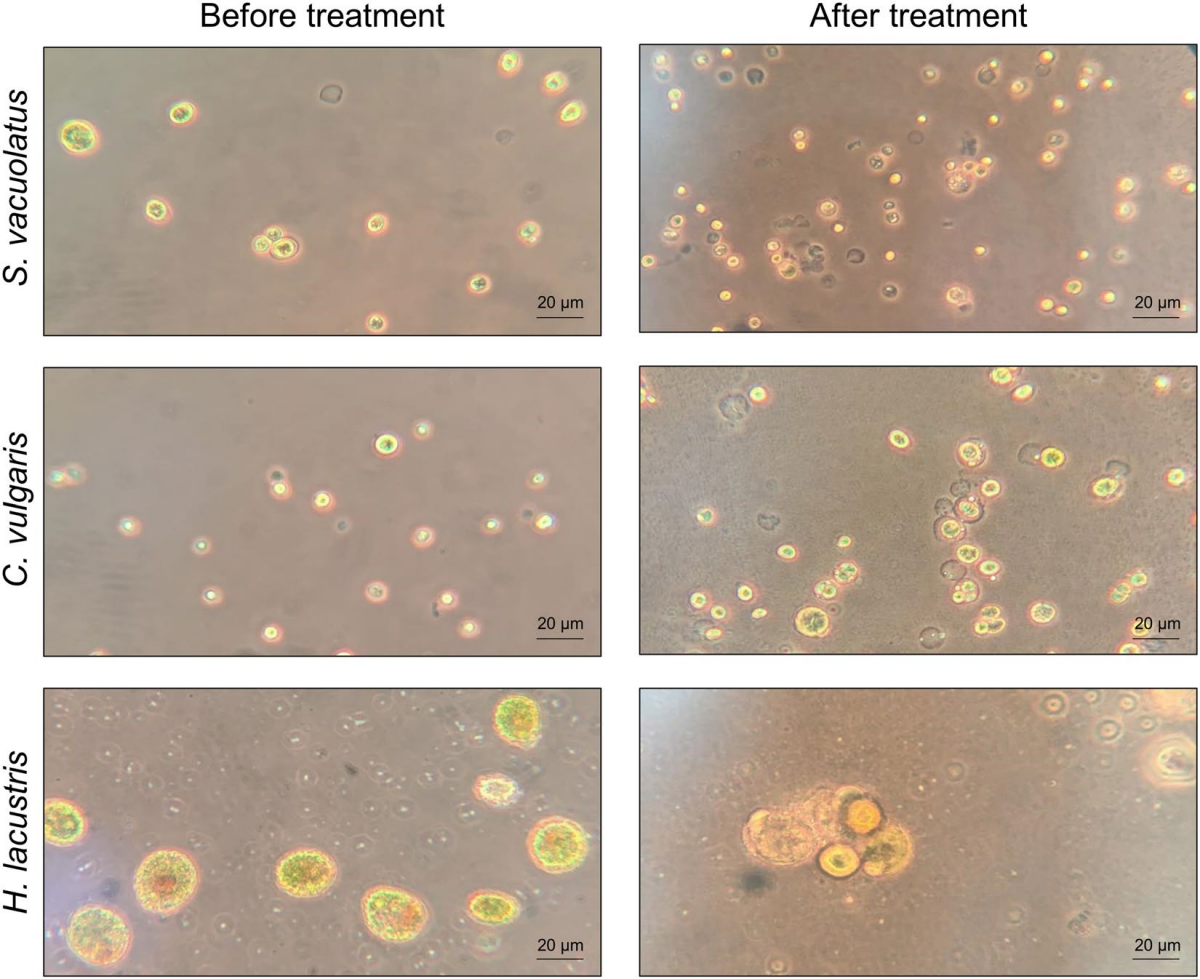
Micrographs of microalgae cells before and after treatment in fluid pyrazine solution. The three microalgae isolates were treated for 6 h and transferred to microscope slides without any further preparations. For all treatments, the bioactive compound was added in a concentration of 1% (v/v).

In order to visualize the effects of alkylpyrazine treatments on the representative microalgae isolates, fluid cultures were treated with 10 µL/mL 5-isobutyl-2,3-dimethylpyrazine for six hours. Microscopic visualization of microalgae cells after treatment showed ruptured cell walls and cell debris in close proximity to algal cytoplasm for all three species (Fig. 5). Moreover, cells with a seemingly intact cytoplasmic membrane and missing cell wall were frequently observed. Most of the cells were visibly impaired by the application of the biocidal compound as indicated by morphological changes when compared to controls that remained untreated. Other effects beside those on the microalgae’s cell walls and cytoplasmic membrane were not observed. The resolution of the obtained micrographs was not sufficient to provide information on changes of intracellular compartments.

## Discussion

This is the first study to demonstrate the biocidal effect of alkylpyrazines on different microalgae species. The efficacy assessment was guided by an analysis of the eukaryotic community and included three representative microalgae isolates for a first evaluation of the compound’s efficacy towards eukaryotic contaminants. Irrespective of the application form, the viability of the implemented test organisms was drastically reduced. Moreover, we observed a complete removal of the model contaminates when a sufficient, isolate-specific concentration of the active compound was applied.

In microalgae cultivations both eukaryotic as well as prokaryotic contaminants can substantially affected the fermentation process. In particular, open pond microalgae cultivation systems are highly susceptible to various kinds of contaminations, which can influence the performance of the desired inoculant^13^. Huo and colleagues identified and isolated the wild strain *Scenedesmus* sp. FS as a contaminant from an outdoor *Chlorella zofingiensis* culture. By showing high alkali resistances and possessing the ability to adapt to various stresses caused by environmental changes, the wild strain was able to quickly replace *C. zofingiensis* and occupy an ecological niche in the photobioreactor^25^. Recent studies also showed that the occurrence of the microalgae *Coelastella* sp. or the co-fermentation of *P. malhamensis*–a member of the phylum *Ochrophyta* – in a microalgae cultivation system can lead to significant biomass yield losses or even the collapse of the main culture^12,26^. These findings reinforce the need for more efficient decontamination of photobioreactors and microalgae cultivation vessels.

In the present study we assessed the efficacy of a volatile antimicrobial alkylpyrazine derivative on three microalgae species - all members of the phylum *Chlorophyta* - which can affect the productivity of the cultivated species due to specific interactions within the community and/or niche occupation^25^. One of the isolates (*Haematococcus* sp.) was the cultivated microalgae in the analyzed process, but was still included in the study as model contaminant, because we found a high number of different OTUs for the same genus. This finding indicated genetic variance of the main inoculum, thus differences in the productivity of the inoculated strain are likely.

While algae accounted for more than 93% of the total eukaryotic population, the second most abundant eukaryotic lineage was assigned to *Protista*, in particular *Amoebozoa*. This group of protists is ubiquitously found in the environment, particularly in freshwater bodies and soil, mainly within biofilms. Some members of this group are known to endure desiccation and harsh environmental conditions for up to 20 years^27,28^. Insufficient cleaning procedures after cultivations may result in an enrichment and manifestation of these organisms due to their enhanced persistence.

Only microalgae were included in this pilot study; however, foregoing studies suggest a broad applicability of certain alkylpyrazines for decontamination purposes. It was shown that the same compound can be employed to reduce highly complex bacterial communities on hatching eggs^21^. The applicability of alkylpyrazines for inactivation of eukaryotic contaminants is supported by the findings of the present study. Here, all tested microalgae were highly susceptible to the bioactive compound irrespective of the treatment approach. An incubation of 30 h was required for a complete deactivation of all tested isolates in fluid cultures when the lowest 5-isobutyl-2,3-dimethylpyrazine concentration was applied. This was mainly due to the resistance of the *S. vacuolatus* isolate at lower concentrations of the antimicrobial compound. In a similar context, *Scenedesmus* sp. LXI has been reported to resist Methylisothiazolinone (MIT), a widely used synthetic biocide in water-containing solutions^29^. Here, the photosynthetic apparatus was damaged by the biocide, but the cell respiration and ATP synthesis remained unaffected. After removal of the biocide, the algae cultures were shown to completely recover. The production of antioxidant enzymes such as superoxide dismutase (SOD) and catalase (CAT) was shown to be crucial for the resistance against MIT-induced damages^29^. By increasing the synthesis of several antioxidant enzymes and non-enzymatic components like β-carotenes or flavonoids, many algae are able to resist reactive oxygen species (ROS) whose formation is induced by various environmental stresses such as extremes of temperature, high salt concentrations, herbicides or UV-radiation^30^. In the present study, increased concentrations and the application of the vaporized pyrazine compound led to a complete elimination of viable cells for all tested microalgae. Notably, the preparation method required for vaporization experiments led to decreased CFU counts in the control treatments that likely resulted from the desiccation-stress that the microalgae were exposed to. Gray and colleagues showed that aquatic microalgae belonging to the family *Chlorophyceae* (e.g. *Scenedesmus* sp.) and *Trebuxiophyceae* (e.g. *Chlorella* sp.) can recover from desiccation after one to five days when rehydrated and re-illuminated^31^. However, the resilience to desiccation differs among different microalgae species^32^.

Microscopic observation of microalgae cells frequently showed impaired cell walls and ruptured cells after alkylpyrazine applications. This is a first indication that pyrazines target the algal cell wall or decrease its stability by direct or indirect interactions. The cell wall composition differs within the employed model species. *Scenedesmus* spp. are known to have pectic layers within their cell wall, which contribute to the stability of the cell wall^33^. The other two species lack this skeletal unit as their cell wall comprises mainly mannose and glucose^33–35^. The observed tolerance of the *Scenedesmus* isolates against the employed alkylpyrazine is potentially linked to structural characteristics of its cell wall. However, the detailed mode of action of bioactive alkylpyrazines against microalgae remains to be elucidated in upcoming studies.

Microalgae are often produced for human consumption and subjected to specific regulations related to food safety^36^. Non-hazardous decontaminations during the production process could improve the quality of the final products by minimizing the occurrence of impurities. In 2002 the Flavor and Extract Manufacturers Association (FEMA) determined pyrazine derivatives to be generally recognized as safe (GRAS) and are considered safe for human consumption at certain intake levels^37^. Their applicability within the food chain  is supported by the fact that various foods naturally harbor specific pyrazine derivatives. Various pyrazines are widely distributed in different vegetables^38^. They occur in trace amounts in numerous plants, where they contribute to the aroma and the characteristic odor of several vegetables^38^. Pyrazine derivatives can further be frequently found in bacteria and in insects where they are assumed to be involved in chemical communication between microorganisms^39–43^. Disinfectants in use such as hydrogen peroxide or sodium hypochlorite have not only negative effects on humans, but also on the environment. Thus, microalgae producers would benefit from new, environmental friendly, natural disinfectants in the cultivation process. In order to make the presented method applicable for industrial decontaminations, extended evaluations with more test organisms and larger sample sizes to validate the promising effects will be required. Further developments of the presented decontamination method could provide a highly efficient alternative for conventional treatments of bioreactors.

## Materials and Methods

### Characterization of microalgae populations in photobioreactors

In order to obtain a complete picture of the eukaryotic community in the photobioreactors of a local producer, we collected samples from reactors that are connected in a multi-stage process. In total eight liquid samples from five different reactors were obtained. The samples were placed on ice and transported in 50 mL plastic tubes to a nearby laboratory. Total community DNA from the reactor samples was extracted using the FastDNA® Kit for Soil (MP Biomedicals, USA), amplified and barcoded with the primer pair 1391 f (5′-GTA CAC ACC GCC CGT C-3′) and EukBr (5′-TGA TCC TTC TGC AGG TTC ACC TAC-3′) targeting the variable region 9 (V9) of the 18S rRNA gene^44^. Each forward and reverse primer contained a specific primer pad (TATGGTAATT/AGTCAGCCAG) and linker (GT/GG), as described in the protocols and standards section of the Earth Microbiome Project (earthmicrobiome.org/)^45^. PCR reactions (20 µL) were executed in triplicates and contained 14.6 µL ultrapure water (Roth, Karlsruhe, Germany), 4 µL Taq&Go (5×; MP Biomedicals, France), 0.2 µL of forward and reverse primer each (10 µM) and 1 µL DNA template (98 °C, 5 min; 10 cycles of 98 °C, 10 sec; 53 °C, 10 sec; 72 °C, 30 sec; 20 cycles of 98 °C, 10 sec; 48 °C, 30 sec; 72 °C, 30 sec; final extension 72 °C, 10 min). PCR products of respective samples had a length of 200 bp and were quality checked by gel electrophoresis. PCR products were purified using Wizard® SV Gel and PCR clean-up system (Promega, Fitchburg, USA) according to manufacturer’s protocol. Purified, barcoded samples were pooled equimolarly and sent for paired-end MiSeq Illumina sequencing (GATC Biotech, Germany). Sequencing data was analyzed using the QIIME 1.9.0 pipeline^46^. Barcodes, primer and adapter sequences were removed and the sequences were quality filtered (maximum unacceptable phred quality score: 19; phred offset: 33). Chimeras were removed from the 18S rRNA gene sequences by using usearch61 to perform both *de novo* (abundance based) and reference based chimera detection. OTU tables were created by an open reference method with UCLUST at a 97% cut-off level for the 18S rRNA gene sequences^47^. OTUs were identified by performing a standard nucleotide BLAST with the NCBI nucleotide collection database excluding uncultured and environmental sample sequences^48^. The final OTU network was constructed by filtering all sequences assigned to *Chlorella*, *Scenedesmus* and *Haematococcus*. Detailed description of QIIME scripts used in bioinformatics analyses are listed in Supplementary Table S4.

### Isolation and identification of microalgae

In order to perform susceptibility tests, the unicellular microalgae *C. vulgaris*, *S. vacuolatus* and *H. lacustris* were isolated from industrial microalgae reactors. Dilution series of respective samples were plated on solid modified Bold’s Basal Medium^49^ (mBBM) containing 250 mg/L NaNO_3_, 175 mg/L KH_2_PO_4_, 75 mg/L K_2_HPO_4_, 75 mg/L MgSO_4_ × 7 H_2_O, 25 mg/L CaCl_2_, 25 mg/L NaCl, 2.6 mg/L H_3_BO_3_, 5 mg/L FeSO_4_ × 7 H_2_O, 8.8 mg/L ZnSO_4_ × 7 H_2_O, 1.4 mgL MnCl_2_ × 4 H_2_O, 1.4 mg/L MoO_3_, 1.6 mg/L CuSO_4_ × 5 H_2_O, 0.5 mg/L Co(NO_3_)_3_ × 6 H_2_O, 0.5 mg/L EDTA, 0.3 mg/L KOH, 0.017 mg/L vitamin B_12_, 0.013 mg/L 4-aminobenzoate, 0.003 mg/L biotin, 0.013 mg/L nicotinic acid, 0.017 mg/L hemicalcium-pentathenate, 0.05 mg/L pyridoxamine-HCl, 0.033 mg/L thiaminiumdichlorid, 0.0091 mg/L thioctic-acid, 0.01 mg/L riboflavin, 0.0049 mg/L folic acid and 18 g/L agar-agar. Vitamins and heat-sensitive components were added after autoclaving by sterile filtration (0.20 µm pore size). In order to obtain pure microalgae cultures, single colonies were picked using a heat sterilized inoculation loop and transferred onto fresh mBBM agar plates. Plates were incubated at room temperature at a light/dark cycle (L:16 h/D:8 h).

In order to identify isolated microalgae species, cells were resuspended in 300 µL 0.85% NaCl and transferred in sterile Eppendorf tubes filled with glass beads. After mechanical disruption using a FastPrep FP120 instrument (MP Biomedicals, Germany) suspensions were centrifuged at 3,000 rpm for 5 min. Supernatant served as template for the following PCR reactions. Partial 18S rRNA gene sequence was amplified by using primer pair TAReuk454FWD1 (5′- CCA GCA SCY GCG GTA ATT CC-3′) and TAReukREV3P (5′-ACT TTC GTT CTT GAT YRA-3′) covering the variable region 4 (V4; 200 bp)^50^. The PCR was performed in a total volume of 30 µL containing 16.2 µL ultrapure water, 6 µL Taq&Go (5×), 2.4 µL MgCl_2_ [25 mM], 1.2 µL of each primer [10 µM] and 3 µL template DNA (98 °C, 30 sec; 10 cycles of 98 °C, 10 sec; 53 °C, 10 sec; 72 °C, 30 sec, followed by 20 cycles of 98 °C., 10 sec; 48 °C, 30 sec; 72 °C, 30 sec; final extension at 72 °C, 10 min). In addition, 18S rRNA gene sequences were amplified using primer pair NS1 (5′-GTA GTC ARA RGC CTT GTC TC-3′) and NS8 (5′-TCC GCA GGT TCA CCT ACG GA-3′)^51^. The PCR was performed in a reaction mix (20 µL) containing 16 µL ultrapure H_2_O, 6 µL Taq&Go (5×), 1.2 µL of each primer [10 µM], 2.4 µL MgCl_2_ [25 mM] and 3 µL DNA template. PCR products were purified using Wizard SV Gel and PCR clean-up system according to manufacturer’s protocol. 18S rRNA gene fragments were sequenced by LGC genomics (Berlin, Germany) and subsequently aligned against the NCBI nucleotide collection database excluding uncultured and environmental sample sequences using the BLAST algorithm^48^.

### Decontamination experiments in fluid suspensions

To obtain pre-cultures for the following experimental procedure, flasks containing 20 mL mBBM were inoculated with a single colony of *Haematococcus* sp., *Scenedesmus* sp. and *Chlorella* sp. respectively and incubated at room temperature at a light/dark cycle (L:16 h/D:8 h) for 5 to 7 days. Following successful cultivation, 1 mL of the pre-culture served as inoculum for 9 mL mBBM. After incubation for 3 days under the above-mentioned conditions, algae suspensions were treated with different concentrations (0.33%, 1.0%, 1.66%) of 5-Isobutyl-2,3-dimethylpyrazine. Experiments were performed in triplicates; for the negative controls equal volumes of 0.85% NaCl were added instead of the pyrazine. Efficiency of treatment was determined by counting CFU on solid mBBM plates that were incubated at room temperature at a light/dark cycle (L:16 h/D:8 h).

### Decontaminations with vaporized alkylpyrazines

Pre-cultures for each tested algae species were obtained by inoculating 30 mL mBBM with single colonies of *H. lacustris*, *S. vacuolatus* and *C. vulgaris* respectively. For *H. lacustris* 5 mL of the pre-culture were further transferred to a 1 L ground flask for 7 days under permanent illumination and aeration. After successful cultivation, the number of living cells in the pre-culture was determined by applying the drop plate technique. For all tested microalgae, 5 mL of the pre-culture suspension were then transferred in sterile 100 mL flasks and dried under sterile conditions. After evaporation of the fluid medium, the flasks were connected to 100 mL flasks filled with 500 µL pyrazine or H_2_O (control); connectors and openings were sealed with paraffin oil. Each of the experiments was performed in triplicates. After 5 h of incubation and vaporization of the alkylpyrazine in the lower flask at 50 °C, the dried algae were rinsed with 2 mL mBBM. The living cell count was determined by using the drop plate technique. After incubation at room temperature at a light/dark cycle (L:16 h/D:8 h) the CFU number was determined for the controls and the pyrazine-treated samples.

### Microscopic visualizations of treated algae

For visualization model microalgae were cultivated in 300 mL flask containing 10 mL sterile mBBM and incubated at room temperature at a light/dark cycle (L:16 h/D:8 h). After a high cell densities were reached (visually assessed), cultures were incubated with 10 µL/mL pyrazine for 6 h. Micrographs of *C. vulgaris*, *S. vacuolatus* and *H. lacustris* cultures before and after treatment were obtained with a light microscope (Leitz; Wetzlar, Germany) at 400× magnification and in combination with a phase contrast objective.

### Statistical analyses

Results presented in the diagrams are the average of three replicates. Statistical analyses were performed using the IBM SPSS program (version 23.0; IBM Corporation, NY, USA). All data was analyzed using Student’s paired t-Test at *p* < 0.01.

## Supplementary information

Supplementary material

## Data Availability

The amplicon data that was used for this study was deposited at ENA (https://www.ebi.ac.uk/ena) under the accession number PRJEB27151.

## References

[1] Plaza M, Herrero M, Cifuentes A, Ibáñez E (2009). Innovative Natural Functional Ingredients from Microalgae. J. Agric. Food Chem..

[2] Chisti Y (2007). Biodiesel from microalgae. Biotechnol. Adv..

[3] Shurtz BK, Wood B, Quinn JC (2017). Nutrient resource requirements for large-scale microalgae biofuel production: Multi-pathway evaluation. Sustain. Energy Technol. Assess..

[4] Pulz O, Gross W (2004). Valuable products from biotechnology of microalgae. Appl. Microbiol. Biotechnol..

[5] Borowitzka MA (2013). High-value products from microalgae—their development and commercialisation. J. Appl. Phycol..

[6] Pulz O (2001). Photobioreactors: production systems for phototrophic microorganisms. Appl. Microbiol. Biotechnol..

[7] Jerney, J. & Spilling, K. Large Scale Cultivation of Microalgae: Open and Closed Systems. *Methods Mol. Biol. Clifton NJ*, 10.1007/7651_2018_130 (2018). 10.1007/7651_2018_130 29480401

[8] Bínová, J., Tichý, V., Lívanský, K. & Zahradník, J. Bacterial contamination of microalgal biomass during outdoor production and downstream processing. *Algol. Stud. Für Hydrobiol. Suppl*. *Vol*. 151–158 (1998).

[9] Letcher PM (2013). Characterization of Amoeboaphelidium protococcarum, an Algal Parasite New to the Cryptomycota Isolated from an Outdoor Algal Pond Used for the Production of Biofuel. PLoS ONE.

[10] Wang L (2016). Contaminating microzooplankton in outdoor microalgal mass culture systems: An ecological viewpoint. Algal Res..

[11] Reich K, Spiegelstein M (1964). Fishtoxins in Ochromonas (chrysomonadina). Isr. J. Zool..

[12] Ma M (2017). Effective control of Poterioochromonas malhamensis in pilot-scale culture of Chlorella sorokiniana GT-1 by maintaining CO2-mediated low culture pH. Algal Res..

[13] Carney, L. T. & Lane, T. W. Parasites in algae mass culture. *Front. Microbiol*. **5** (2014). 10.3389/fmicb.2014.00278 PMC4047527 24936200

[14] Wang B, Lan CQ, Horsman M (2012). Closed photobioreactors for production of microalgal biomasses. Biotechnol. Adv..

[15] Singh RN, Sharma S (2012). Development of suitable photobioreactor for algae production–A review. Renew. Sustain. Energy Rev..

[16] Wang H, Zhang W, Chen L, Wang J, Liu T (2013). The contamination and control of biological pollutants in mass cultivation of microalgae. Bioresour. Technol..

[17] Klapes NA, Vesley D (1990). Vapor-phase hydrogen peroxide as a surface decontaminant and sterilant. Appl. Environ. Microbiol..

[18] Johnston MD, Lawson S, Otter JA (2005). Evaluation of hydrogen peroxide vapour as a method for the decontamination of surfaces contaminated with Clostridium botulinum spores. J. Microbiol. Methods.

[19] Rybakova D (2017). Aerial Warfare: A Volatile Dialogue between the Plant Pathogen Verticillium longisporum and Its Antagonist Paenibacillus polymyxa. Front. Plant Sci..

[20] Cernava T, Aschenbrenner IA, Grube M, Liebminger S, Berg G (2015). A novel assay for the detection of bioactive volatiles evaluated by screening of lichen-associated bacteria. Front. Microbiol..

[21] Kusstatscher P, Cernava T, Liebminger S, Berg G (2017). Replacing conventional decontamination of hatching eggs with a natural defense strategy based on antimicrobial, volatile pyrazines. Sci. Rep..

[22] Schöck M, Liebminger S, Berg G, Cernava T (2018). First evaluation of alkylpyrazine application as a novel method to decrease microbial contaminations in processed meat products. AMB Express.

[23] AICHNER, M. *et al*. Volatile organic compounds from bacterial antagonists for controlling microbial growth (2013).

[24] Fürnkranz M (2012). Microbial diversity inside pumpkins: microhabitat-specific communities display a high antagonistic potential against phytopathogens. Microb. Ecol..

[25] Huo S (2017). Outdoor Growth Characterization of an Unknown Microalga Screened from Contaminated *Chlorella* Culture. BioMed Res. Int..

[26] Dawidziuk A (2017). Assessing contamination of microalgal astaxanthin producer Haematococcus cultures with high-resolution melting curve analysis. J. Appl. Genet..

[27] Balczun C, Scheid PL (2018). Lyophilisation as a simple and safe method for long-term storage of free-living amoebae at ambient temperature. Parasitol. Res..

[28] Sriram R, Shoff M, Booton G, Fuerst P, Visvesvara GS (2008). Survival of Acanthamoeba Cysts after Desiccation for More than 20 Years. J. Clin. Microbiol..

[29] Wang X-X, Zhang T-Y, Dao G-H, Hu H-Y (2018). Tolerance and resistance characteristics of microalgae Scenedesmus sp. LX1 to methylisothiazolinone. Environ. Pollut. Barking Essex.

[30] Mallick N, Mohn FH (2000). Reactive oxygen species: response of algal cells. J. Plant Physiol..

[31] Gray DW, Lewis LA, Cardon ZG (2007). Photosynthetic recovery following desiccation of desert green algae (Chlorophyta) and their aquatic relatives. Plant Cell Environ..

[32] Wieners PC, Mudimu O, Bilger W (2018). Survey of the occurrence of desiccation-induced quenching of basal fluorescence in 28 species of green microalgae. Planta.

[33] Bisalputra T, Weier TE (1963). The Cell Wall of Scenedesmus quadricauda. Am. J. Bot..

[34] Loos E, Meindl D (1982). Composition of the cell wall of Chlorella fusca. Planta.

[35] Hagen C, Siegmund S, Braune W (2002). Ultrastructural and chemical changes in the cell wall of Haematococcus pluvialis (Volvocales, Chlorophyta) during aplanospore formation. Eur. J. Phycol..

[36] Enzing, C., Ploeg, M., Barbosa, M. & Sijtsma, L. *Microalgae-based products for the food and feed sector: an outlook for Europe*. 10.2791/3339 (Publications Office of the European Union, 2014).

[37] Adams TB (2002). The FEMA GRAS assessment of pyrazine derivatives used as flavor ingredients. Flavor and Extract Manufacturers Association. Food Chem. Toxicol. Int. J. Publ. Br. Ind. Biol. Res. Assoc..

[38] Murray KE, Whitfield FB (1975). The occurrence of 3-alkyl-2-methoxypyrazines in raw vegetables. J. Sci. Food Agric..

[39] Bramwell, F. A., Burrell, K. J. W. & Riezebos, G. Characterisation of pyrazines in Galbanum oil. | Article Information | J-GLOBAL. *Tetrahedron Lett* 3215–3216 (1969).

[40] Buttery RG, Seifert RM, Guadagni DG, Ling LC (1969). Characterization of some volatile constituents of bell peppers. J. Agric. Food Chem..

[41] Beck HC, Hansen AM, Lauritsen FR (2003). Novel pyrazine metabolites found in polymyxin biosynthesis by Paenibacillus polymyxa. FEMS Microbiol. Lett..

[42] Rybakova D (2016). Endophytes-assisted biocontrol: novel insights in ecology and the mode of action of Paenibacillus. Plant Soil.

[43] Vander Meer RK, Preston CA, Choi M-Y (2010). Isolation of a pyrazine alarm pheromone component from the fire ant, Solenopsis invicta. J. Chem. Ecol..

[44] Amaral-Zettler LA, McCliment EA, Ducklow HW, Huse SM (2009). A Method for Studying Protistan Diversity Using Massively Parallel Sequencing of V9 Hypervariable Regions of Small-Subunit Ribosomal RNA Genes. PLOS ONE.

[45] Thompson LR (2017). A communal catalogue reveals Earth’s multiscale microbial diversity. Nature.

[46] Caporaso JG (2010). QIIME allows analysis of high-throughput community sequencing data. Nat. Methods.

[47] Edgar RC (2010). Search and clustering orders of magnitude faster than BLAST. Bioinforma. Oxf. Engl..

[48] Altschul SF, Gish W, Miller W, Myers EW, Lipman DJ (1990). Basic local alignment search tool. J. Mol. Biol..

[49] Bold HC (1949). The Morphology of Chlamydomonas chlamydogama, Sp. Nov. Bull. Torrey Bot. Club.

[50] Stoeck T (2010). Multiple marker parallel tag environmental DNA sequencing reveals a highly complex eukaryotic community in marine anoxic water. Mol. Ecol..

[51] White, T. J., Bruns, T., Lee, S. & Taylor, J. Amplification and direct sequencing of fungal ribosomal RNA genes for phylogenetics. In *PCR Protocols* **18**, 315–322 (Academic Press, 1990).

