# Supplementary material for: A novel, nature-based alternative for photobioreactor decontaminations
Source: Sci Rep. 2019 Feb 27;9:2864. doi: 10.1038/s41598-019-39673-6 (PMC6393562; doi:10.1038/s41598-019-39673-6)
Supplement: Supplementary file 1 — Supplementary material [file 41598_2019_39673_MOESM1_ESM.docx]

**Supplementary Data**

**A novel, nature-based alternative for photobioreactor decontaminations**

**Lisa Krug**^1^**^,2^, Armin Erlacher^2^, Gabriele Berg^2^, and Tomislav Cernava^2*^**

*^1^ACIB GmbH, Petersgasse 14, 8010 Graz, Austria*

*^2^Institute of Environmental Biotechnology, Graz University of Technology, Petersgasse 12, 8010 Graz, Austria*

**Correspondence:*

*Tomislav Cernava*

*Institute of Environmental Biotechnology,Graz University of Technology, Petersgasse 12, 8010 Graz, Austria.*

*e-mail:* [*tomislav.cernava@tugraz.at*](mailto:gabriele.berg@tugraz.at)

**Running title:** Pyrazine-based bioreactor decontamination

**Submitted to:** Scientific Reports

**Keywords:** alkylpyrazines; microalgae reactor; bioreactor; decontamination; antimicrobial compounds

**Table S1.** **Closest hits of OTUs represented by more than 100 reads.** Reference sequences were aligned with NCBI’s standard nucleotide BLAST against the NCBI nucleotide collection database excluding uncultured and environmental sample sequences.

| **OTU ID** | **Closest NCBI hit** | **Accession number** | **Identity** | **Read count** |
| --- | --- | --- | --- | --- |
| OTU 22 | *Haematococcus lacustris* 18S ribosomal RNA gene | KY364700.1 | 99 % | 4,367,178 |
| OTU 2041 | *Haematococcus lacustris* strain KMMCC 1552 18S ribosomal RNA gene | JQ315538.1 | 100 % | 143,109 |
| OTU 29 | *Haematococcus lacustris* 18S ribosomal RNA gene | KY364700.1 | 97 % | 713 |
| OTU 3067 | *Haematococcus lacustris* 18S ribosomal RNA gene | KY364700.1 | 92 % | 201 |
| OTU 1641 | *Haematococcus lacustris* 18S ribosomal RNA gene | KY364700.1 | 99 % | 196 |
| OTU 30 | *Chlorella vulgaris* isolate 18s rRNA small subunit ribosomal RNA gene | MF686452.1 | 100 % | 35,252 |
| OTU 1748 | *Chlorella* sp. ZJU0201 18S ribosomal RNA gene | JX097053.1 | 100 % | 2,451 |
| OTU 3055 | *Chlorella* sp. SAG 222-2a 18S rRNA gene (partial) | FM205857.1 | 100 % | 171 |
| OTU 4 | *Scenedesmus vacuolatus* 18S small subunit rRNA | X56104.1 | 99 % | 1,831 |

**Table S2.** **CFU/mL counts following treatments in liquid alkylpyrazine solutions.** Different incubation times were tested in combination with three distinct pyrazine concentrations. A control without supplementation of the bioactive compound was included for comparisons.

|  | **time**  **[h]** | **control**  **[×10^5^ CFU/mL ]** | **3.3 µL/mL**  **[×10^5^ CFU/mL]** | **10.0 µL/mL**  **[×10^5^ CFU/mL]** | **16.6 µL/mL**  **[×10^5^ CFU/mL]** |
| --- | --- | --- | --- | --- | --- |
| ***S. vacuolatus*** | 0 | 2.64 ± 0.17 | 2.83 ± 0.37 | 2.93 ± 0.34 | 2.23 ± 0.19 |
|  | 2 | 2.84 ± 0.65 | 0.05 ± 0.03 | 0.00 ± 0.00 | 0.00 ± 0.00 |
|  | 4 | 2.41 ± 0.52 | 0.03 ± 0.02 | 0.00 ± 0.00 | 0.00 ± 0.00 |
|  | 6 | 2.41 ± 0.89 | 0.01 ± 0.00 | 0.00 ± 0.00 | 0.00 ± 0.00 |
|  | 30 | 2.44 ± 0.22 | 0.00 ± 0.00 | 0.00 ± 0.00 | 0.00 ± 0.00 |
| ***C. vulgaris*** | 0 | 55.3 ± 8.02 | 53.3 ± 9.78 | 46.2 ± 6.67 | 58.3 ± 2.50 |
|  | 2 | 42.3 ± 19.3 | 0.00 ± 0.00 | 0.00 ± 0.00 | 0.00 ± 0.00 |
|  | 4 | 58.1 ± 22.8 | 0.00 ± 0.00 | 0.00 ± 0.00 | 0.00 ± 0.00 |
|  | 6 | 54.8 ± 2.40 | 0.00 ± 0.00 | 0.00 ± 0.00 | 0.00 ± 0.00 |
|  | 30 | 57.9 ± 16.8 | 0.00 ± 0.00 | 0.00 ± 0.00 | 0.00 ± 0.00 |
| ***H. lacustris*** | 0 | 0.24 ± 0.03 | 0.25 ± 0.02 | 0.20 ± 0.05 | 0.33 ± 0.05 |
|  | 2 | 0.34 ± 0.18 | 0.00 ± 0.00 | 0.00 ± 0.00 | 0.00 ± 0.00 |
|  | 4 | 0.32 ± 0.15 | 0.00 ± 0.00 | 0.00 ± 0.00 | 0.00 ± 0.00 |
|  | 6 | 0.45 ± 0.04 | 0.00 ± 0.00 | 0.00 ± 0.00 | 0.00 ± 0.00 |
|  | 30 | 0.48 ± 0.35 | 0.00 ± 0.00 | 0.00 ± 0.00 | 0.00 ± 0.00 |

**Table S3.** **Total CFU/ml counts following the treatment with vaporized 5-isobutyl-2,3-dimethylpyrazine and an incubation time of 5 h.**

| **Organism** | **Pre-culture**  **[×10^5^CFU/mL]** | **Control**  **[×10^5^CFU/mL]** | **Treatment**  **[×10^5^CFU/mL]** |
| --- | --- | --- | --- |
| ***S. vacuolatus*** | 57.4 ± 4.49 | 0.11 ± 0.09 | 0.00 ± 0.00 |
| ***C. vulgaris*** | 39.6 ± 7.09 | 31.7 ± 3.60 | 0.00 ± 0.00 |
| ***H. lacustris*** | 20.5 ± 5.84 | 4.10 ± 2.10 | 0.00 ± 0.00 |

**Table S4: QIIME scripts used for bioinformatics analyses of the amplicon dataset.** For network rendering and diversity analyses, the OTUs were manually identified with nucleotide BLAST searches within the NCBI nucleotide collection database.

| **Process step** | **Script** | **Pipeline, plugins and parameters** |
| --- | --- | --- |
| Join reads | join_paired_ends.py | QIIME 1.9.0 (SeqPrep) |
| Remove barcodes from sequences | extract_barcodes.py | QIIME 1.9.0 |
| Assign sequences to samples / Check quality | split_libraries_fastq.py | QIIME 1.9.0 (quality score: 19; phred offset: 33) |
| Identify chimeric sequences | identify_chimeric_seqs.py | QIIME 1.9.0 / usearch61 |
| Remove chimeric sequences | filter_fasta.py | QIIME 1.9.0 |
| Identify OTUs | pick_open_reference_otus.py | QIIME 1.9.0 / UCLUST (97% cutoff level) / DB: SILVA release 119 |
| Compute node and edge table for OTU network | make_otu_network.py | QIIME 1.9.0 |
